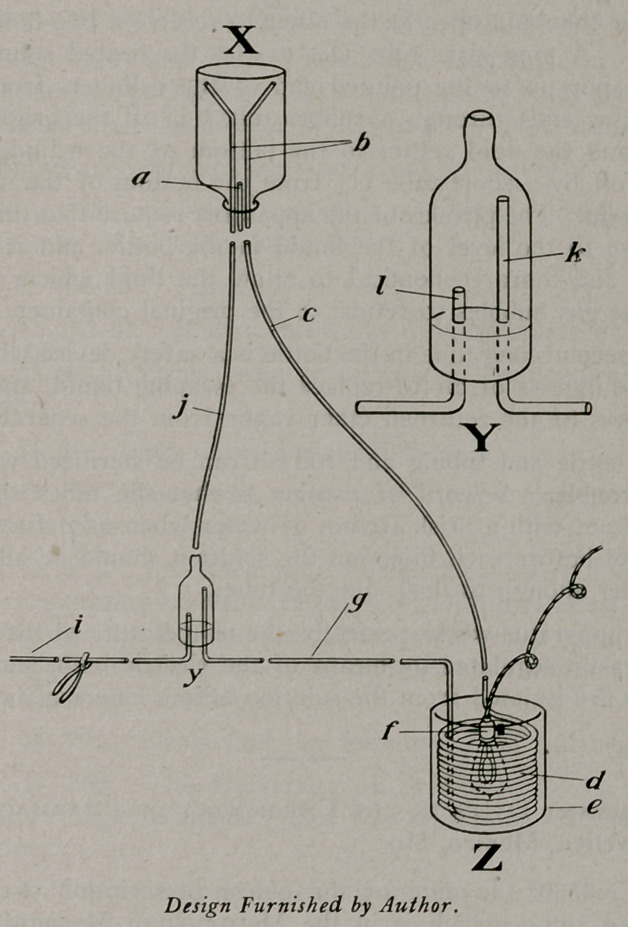# General Anaesthesia by Intravenous Injection of Ether-Salt Solution

**Published:** 1913-09

**Authors:** 


General Anaesthesia by Intravenous Injection of Ether-salt Solution, Dr. L. W. Jenkins, Asst. Surg. U. S. P. H. Service, San Francisco, *Northwest Medicine,* December, 1912. (Cut loaned by courtesy of Editor.)

To avoid danger of contamination through repeated handling of the solution, we have come to use a large bottle marked at 2325 cc. and at 2500 cc. Into this bottle is inserted a three-hole

*Design Furnished by Author.*

rubber stopper with a short glass tube (a) passing barely through the stopper, and two long glass tubes (b) reaching to the bottom of the bottle. Attached to the short glass tube is 125 cm. of rubber tubing (c) 5 mm. in diameter, passing to a copper tube coil (d) of the same caliber, 300 cm. in length. This copper coil fits into a basin (e) 15 cm. in diameter of about a litre capacity. This basin is filled above the coil with water kept at a temperature of

45° C. by a 32 cp. electric bulb (f). Another rubber tube (g) leads from the copper coil to what we may call a "gas separator" (y) which should be in immediate proximity to the canula (i) inserted into the vein. From the separator another piece of rubber tubing (j) 3mm. caliber returns to the bottle and is attached to one of the glass tubes extending to the bottom of the bottle.

The "gas separator" is a simple but important piece of apparatus consisting of a 2cm. glass cylinder pointed at one end to receive a rubber tube and open at the other to receive a two-hole rubber stopper. A long glass tube (k) carries the heated solution and ether vapor up to the pointed end of the cylinder, from which the gas ascends through a rubber tube (j) to the original container and the fluid settles to the bottom of the cylinder, being drawn off by a short tube (l) from the bottom of the separator to the vein. The physics of the apparatus require that this return tube rise to the level of the liquid in the bottle, and it is more cleanly and more economical to allow the fluid which is raised with the gas bubbles to return to the original container.

The second long tube in the bottle is a safety device which permits the ingress of air to replace the escaping liquid, and allows the egress of the returned ether vapor from the separator.

The bottle and tubing and coil all can be sterilized with very little trouble. A word of caution is that the tubes should be washed out with a brisk stream of water when used for the first time and before each injection the solution should be allowed to flow long enough to flush out the tubes.

This apparatus works perfectly, the temperature of the solution being easily regulated by means of the electric bulb, and all gas bubbles are isolated from the solution before entering the vein.

## Figures and Tables

**Figure f1:**